# Evaluation of *CX3CR1* gene DNA methylation in developmental dysplasia of the hip (DDH)

**DOI:** 10.1186/s13018-022-03324-w

**Published:** 2022-09-29

**Authors:** Mohammad Nejadhosseinian, Hoda Haerian, Reza Shirkoohi, Jafar Karami, Seyed Mohammad Javad Mortazavi

**Affiliations:** 1 Joint Reconstruction Research Center, Tehran University of Medical Sciences, Tehran, Iran; 2 Rheumatology Research Center, Tehran University of Medical Sciences, Tehran, Iran; 3 Department of Medical Genetics, Cancer institute, Tehran University of Medical Sciences, Tehran, Iran; 4 Molecular and Medicine Research Center, Khomein University of Medical Sciences, Khomein, Iran; 5 Department of Laboratory Sciences, Khomein University of Medical Sciences, Khomein, Iran; 6 Department of Orthopedic Surgery, Tehran University of Medical Sciences, Tehran, Iran

**Keywords:** *CX3CR1* gene, DNA methylation, Developmental dysplasia of the hip (DDH)

## Abstract

**Introduction and objective:**

Developmental dysplasia of the hip (DDH) is a musculoskeletal disorder. Genetic and epigenetic changes in C-X3-C motif chemokine receptor 1 (*CX3CR1*) may lead to disturbance in chondrocyte development and change the labrum dimensions, which indirectly result in hip joint instability. Considering the important role of this gene in cell migration, cell adhesion and bone and cartilage development, we aimed to evaluate the *CX3CR1* gene methylation in DDH pathogenesis.

**Methods:**

Our study comprised of forty-five DDH patients and forty-five healthy control subjects with healthy femoral neck cartilage. The healthy controls had total or hemiarthroplasty for the femoral neck fracture. Samples were collected from the femoral head (cartilage) of DDH patients and healthy controls. Genomic DNA was obtained from the samples, and DNA methylation of *CX3CR1* gene was analyzed via metabisulfite method.

**Results:**

Methylation analysis reveals no significant differences in promoter of *CX3CR1* gene in cartilage samples from DDH patients and healthy control subjects (*P* = 0.33).

**Conclusion:**

Methylation status of *CX3CR1* gene showed no significant difference between the patient and control groups. Our results indicate that DNA methylation may not modulate this gene in this disease and other epigenetic mechanisms such as non-coding RNAs and histone modifications could be implicated.

## Introduction

Developmental dysplasia of the hip (DDH) is one of the musculoskeletal disorder that is common in infancy and shows clinical manifestations ranging from clicky or stable hips with ultrasound or radiological evidence of acetabular dysplasia to dislocated, subluxatable or dislocatable hips [1, 2]. The normal development of the hip is dependent on a normal development of both the femoral head and the acetabulum. The femoral head must be firm and stable in the hip socket to form spherically and concentrically. In a situation with loose interaction between the acetabulum and the femoral head or with deficiency of either component, the outcome would be lack of sphericity and incongruence for hip joint [3]. In DDH, hips could be dislocated, subluxated, malformed or unstable. Dislocation includes displacement of the femoral head from the acetabulum completely. Subluxation indicates dislocation between the femoral head and acetabulum with some contact. Malformation is any abnormality in the development of the acetabulum and/or femoral head and instability includes inability of the hip to resist an externally applied force without developing a dislocation or subluxation [1]. In cases with mild dysplasia, the symptoms may not have presented clinically at all or may present itself at adult life but in cases with severe dysplasia the symptoms are present clinically in early childhood or infancy [4]. The incidence of DDH is almost 1 in 1000 live births. Since there is a lack of uniformity for DDH definition, the true incidence is not known, but the incidence of entire spectrum of DDH is much higher [3, 5–7]. Actually, the DDH incidence is not constant and relays on many factors. These inconsistency comes from the variables such as DDH definition, differences in clinical methods and skills for detection, timing of evaluation and genetic differences [8, 9]. Furthermore, gender could play a part, since DDH is more common in girls than boys [8]. Generally, the risk factors for DDH include mechanical constriction of the fetus, abnormal position in the 3rd trimester, postnatal environment and genetic risk factors [10, 11]. The risk factors connected to intrauterine mechanical constraint are large birth weight breech presentation and oligohydramnios [8, 12, 13].

A risk factor that is recently reported for DDH development is epigenetics. Since the concordance rate in monozygotic twin for DDH development is approximately 40%, the remaining risk factors are environmental factors such as epigenetics. DDH could be considered as a multifactorial disease with genetic and non-genetic risk factors that are implicated in pathogenesis of DDH [14–16]. Epigenetic is a process which affects and regulates gene expression through different mechanisms such as non-coding RNAs, histone modifications and DNA methylation. DNA methylation is the most important epigenetic mechanisms which modulates gene expression through heterochromatin and euchromatin formation [17]. We previously reported that the growth/differentiation factor 5 (*GDF5)* gene is hypermethylated in DDH patients [18].

C-X3-C motif chemokine receptor 1 (CX3CR1) is a chemokine and transmembrane protein involved in the migration and adhesion of leukocytes. It is documented that this receptor has vital role in homeostasis of bone and cartilage. Genetic and epigenetic changes in *CX3CR1* may lead to disturbance in development of chondrocyte and change the labrum dimensions which indirectly result in hip joint instability [19]. Furthermore, Hoshino and colleagues have revealed that CX3CR1-CX3CL1 interactions play critical roles differentiation of osteoclasts and osteoblasts [20]. Another study by Djouad and colleagues illustrated that human mesenchymal stem cells are expressing high level of CX3CR1 in comparison to chondrocytes [21]. These results suggest that the mesenchymal cells require chemokine receptors for receiving signals and migration and these receptors may implicate in the developing structure of the cartilage anlage of the acetabulum.

We aimed to evaluate the methylation status of the *CX3CR1* gene in DDH pathogenesis. According to literature review, this is the first study that provides insight about methylation pattern of *CX3CR1* gene in DDH pathogenesis.

## Material and methods

### DDH patients and healthy controls

DDH patients were consecutively recruited between 2017 and 2018 from the orthopedics clinic of Imam Khomeini Hospital of Tehran University of Medical Sciences. The healthy subjects are participated in the study at the same time. Diseases such as osteoarthritis, metabolic bone disease and DDH were excluded from the control group. Our study comprises of 90 individuals (45 healthy controls and 45 DDH patients), and the mean age ± SD of controls and patients was 42 ± 15.2 and 45 ± 12.6, respectively. The male-to-female ratio was equal in patient and control groups (5 males and 40 females). The control individuals had neither family history nor clinical evidence of arthritis or any inflammatory disorders. The control samples were taken from people who were under a hemi- or total arthroplasty operation because of femoral head fracture. These cartilage samples are actually obtained from individuals with healthy femoral head. The Human Research Ethics Committee of Tehran University of Medical Sciences approved this study (IR.TUMS.IKHC.REC.1397.179). Informed consent was obtained from all control and patient subjects.

### Sample collection

Samples were collected from the femoral head cartilage of healthy control subjects and DDH patients. QIAamp DNA Mini Kit (Qiagen) was used to extract genomic DNA from the samples according to the manufacturer’s instruction. DNA from all the samples extracted and stored at − 20 °C. Purity and yield of DNA were determined using a NanoDrop spectrophotometer at 260 nm and 280 nm (NanoDrop 2000c Spectrophotometer, Thermo Fisher Scientific, Wilmington, DE, USA).

### Bisulfite treatment

The extracted DNA was treated with bisulfite (EpiTect Plus DNA Bisulfite Kit). The principle of this method of treatment is that the methylated cytosines will remain unchanged, while the unmethylated cytosine will be converted to uracil. According to bisulfite treatment kit, the following notes were applied before starting.

30 ml of 96% ethanol was added to Buffer BW and stored at room temperature (15–25 °C). 27 ml of 96% ethanol is added to buffer BD and stored at 2–8 °C. 310 μl of RNase-free water is added to carrier RNA and stored at − 20 °C.

### EpiTect Plus DNA Bisulfite Kit

800 μl of RNase-free water was added to each aliquot of Bisulfite Mix and then vortexed to completely dissolve the Bisulfite Mix (several minutes). 200 μl PCR tubes were used for bisulfite reactions based on the protocol. The tubes were closed and mixed for several minutes. The blue color of the DNA Protect Buffer is considered as a correct PH and sufficient mixture. Thermal cycler program was set up, the tubes were located in the thermal cycler, and the incubation time for each section was obtained from the protocol.

After PCR, the tubes were centrifuged and the reactions were transferred into microcentrifuge clean tubes (1.5 ml), and then, 310 μl of Buffer BL was added into each tube according to the protocol, mixed and centrifuged shortly. Afterward, 250 μl of 96% ethanol was added into each tube, mixed, vortexed and centrifuged.

Then, the reactions from tubes were transferred into spin columns which were located on collection tubes and centrifuged for 60 s. The flow-through was discarded from collection tubes, and spine tubes were placed on collection tubes again.

The Buffer BW (500 μl) was added into each spine column, and the tubes were centrifuged for 60 s. Again, the flow-through was discarded from collection tubes and spine tubes were placed into collection tubes.

The Buffer BD (500 μl) was added into each spin column and incubated at room temperature for 15 min and then centrifuged for 60 s. Afterward, the flow-through was discarded from collection tubes and spine tubes were placed into collection tubes.

The Buffer BW (500 μl) was added into each spin column and centrifuged for 60 s. Then, the flow-through was discarded from collection tubes and spine tubes were placed into collection tubes. (This step was repeated again.)

The 96% ethanol (250 μl) was added into each spin column and centrifuged for 60 s. Then, spin columns were placed into collection tubes (2 ml) and centrifuged for 60 s. In order to evaporate the liquid, the tubes were incubated on a heating block for 5 min at 60 °C.

The spin columns were placed into microcentrifuge clean tubes (1.5 ml). Then, the Buffer EB (15 μl) was added into each spin column, incubated at room temperature for 60 s. To elute the DNA, tubes were centrifuged. Finally, the purified DNA was stored for up to 24 h at 2–8 °C. In order to use samples for more works and further evaluation, DNA should be stored at − 20 °C.

### PCR amplification

A 221 base-pair segment of DNA at 3p22 band was target for PCR amplification. The segment was part of the promoter region of *CX3CR1* gene. The DNA sequence of *CX3CR1* promoter was obtained from the UCSC (University of California, Santa Cruz) website (https://genome.ucsc.edu/). The bisulfite specific primers for PCR were designed using MethPrimer website (http://www.urogene.org/cgi-bin/methprimer/methprimer.cgi).

Each PCR tube contained DNA Protect Buffer (35 μl), bisulfite mix (85 μl), RNase-free water (variable) and bisulfite-treated DNA (maximum 20 μl). For samples with high concentration (ranged from 1 ng to 2 μg), the final volume of RNase-free water and DNA solution was 20 μl. The PCR conditions were as follows: denaturation (95 °C for 5 min), incubation (60 °C for 25 min), denaturation (95 °C for 5 min), incubation (60 °C for 85 min), denaturation (95 °C for 5 min) and incubation (60 °C for 175 min). For amplification validation, all DNA samples were gel electrophoresed after PCR amplification with 2% agarose gel. After confirmation with electrophoresis, the samples were sequenced (Macrogen, Seoul, Korea) and evaluated using Codon Code Aligner version 2 (Codon Code Corporation, Dedham, MA, USA) software.

### Statistical analysis

Our data were analyzed with SPSS version 22.0, and graph was generated using GraphPad Prism version 5 for windows (GraphPad Software, La Jolla, CA USA, www. graphpad.com). All data are shown as mean ± standard deviation (S.D.) and also analyzed for normal distribution using the Kolmogorov–Smirnov test. Mann–Whitney test and independent sample t test were applied. Value less than 0.05 was considered statistically significant.

## Results

### Methylation status of *CX3CR1*

**Fig. 1:**
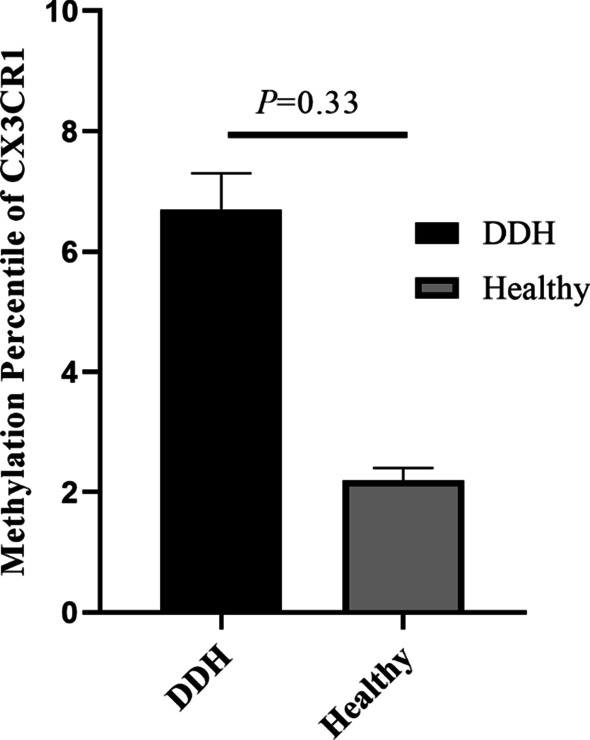
Methylation status of *CX3CR1* gene promoter in DDH patients and healthy controls

Methylation level of *CX3CR1* gene in healthy control subjects and DDH patients is illustrated in Table 1 and Fig. 1. Methylation analysis reveals no significant differences in promoter of *CX3CR1* gene between healthy control subjects and DDH patients (*P* = 0.33, Fig. 1, Table 1).

**Table 1:** Methylation status of *CX3CR1* gene promoter in DDH patients and healthy controls

*CX3CR1*	Groups		*P*-value
	DDH	Healthy	
Methylated	3 (6.7%)	1 (2.2%)	0.33
Unmethylated	42 (93.3%)	44 (97.8)	
Total	45 (100%)	45 (100%)

## Discussion

DDH is a multifactorial disease that genetic factors and non-genetic factors such as epigenetics are implicated in its etiopathogenesis [3]. Monozygotic and dizygotic twin studies demonstrated that genetic factors are involved in the disease, but also indicated that there are others susceptible factors such as epigenetic and environmental factors [22, 23]. Actually, DDH is a complex disorder and genetic and epigenetic factors are contributed to its pathogenesis. Recently, researchers are interested toward the interaction and collaboration between environmental and genetic factors which finally modify the epigenome [24, 25]. In order to determine the DDH etiology, epigenetic mechanisms such as DNA methylation have a unique importance.

According to our knowledge, we could not find any study evaluating the methylation status of *CX3CR1* gene in DDH pathogenesis, while there are some studies showing that the *CX3CR1* could be considered as a genetic susceptible risk factor for DDH [26–29]. On the other hand, there are studies demonstrating that the *CX3CR1* gene could be regulated through DNA methylation in human glioma tumors [30], myeloid cells [31] and CD8^+^ T cells [32].

These studies propose that the *CX3CR1* gene could be a susceptible risk factor for DDH and also could be regulated through DNA methylation. Nonetheless, there is no report of evaluating methylation status of *CX3CR1* in DDH. Our study showed no significant difference in methylation level of *CX3CR1* gene between DDH patients and healthy controls. Our results indicate that this gene may not be regulated through DNA methylation mechanism in this disease and could be regulated via other epigenetic mechanisms such as microRNAs and histone modifications.

There are several issues which need to be addressed. In case of DNA methylation, it is not completely clear that DNA methylation abnormalities, which are reported through different studies [25, 33–37], are actually consequence of the disease or could be considered as a disease causes. Since the DNA methylation could be affected by disease stage and chronic inflammatory situation of disease, it seems that these abnormalities are a disease consequence rather than the disease cause. In order to understand the role of DNA methylation in disease pathogenesis, we have to compare the methylation status of a gene or genome at the early and late stage of diseases.

In summary, epigenetic modifications such as non-coding microRNAs, histone modifications, and DNA methylation could be considered as a diagnostic and prognostic marker in DDH. These alterations could be a promising therapeutic approach in near future for DDH patients. Fortunately, our understanding about epigenetic changes in DDH has been improved and gives us a chance to prevent or control the disease. Although methylation may not participate in modulating *CX3CR1* gene in DDH pathogenesis, role of other epigenetic mechanisms should be explored.

## Data Availability

Data are available upon request.
